# Construction of Ecological Security Patterns in Nature Reserves Based on Ecosystem Services and Circuit Theory: A Case Study in Wenchuan, China

**DOI:** 10.3390/ijerph16173220

**Published:** 2019-09-03

**Authors:** Jianying Xu, Feifei Fan, Yanxu Liu, Jianquan Dong, Jixing Chen

**Affiliations:** 1 College of Resource, Environmental and Tourism, Capital Normal University, Beijing 100048, China (J.X.) (F.F.) (J.C.); 2 State Key Laboratory of Earth Surface Processes and Resource Ecology, Faculty of Geographical Science, Beijing Normal University, Beijing 100875, China; 3 Laboratory for Earth Surface Processes, Ministry of Education, College of Urban and Environmental Sciences, Peking University, Beijing 100871, China

**Keywords:** ecological security patterns, ecosystem services, circuit theory, landscape transformation, nature reserves

## Abstract

Facing the demands of biodiversity conservation and ecosystem service improvement, the spatial pattern optimization of nature reserves has always been a research topic of interest. However, there remains a lack of methodological guidance in the planning of nature reserves and the surrounding areas. To promote the landscape sustainability of nature reserves, we constructed ecological security patterns (ESPs) with two scenarios as a case study in Wenchuan, China. In detail, the ecological sources were identified by ecosystem service evaluation, and the resistance surface was characterized by the habitat quality. The ecological corridors were determined based on circuit theory and the minimum cumulative resistance model. The ecological sources were mainly aggregated in the protected areas, with an area of more than 1000 ha; the high-resistance values were mainly in the area with dense roads or high elevation. There were 21 corridors in the scenario of only optimizing the nature reserve, while 31 corridors were identified when considering non-nature reserves, and the landscape connectivity was enhanced accordingly. The result supported constructing the ESPs between nature and non-nature reserves in Wenchuan to further protect pandas, and a methodological contribution was made to understand the differences of ESPs between them, thus supporting a methodological formulation of sustainable landscape patterns.

## 1. Introduction

In the process of regional development, population growth and economic development have led to tremendous pressure on the ecosystem, causing degradation such as water shortages, soil erosion, carbon loss, biodiversity loss, and fragmentation [1,2,3,4]. Facing the relationship of nature and human beings, sustainability has been regarded as an adaptive process that can maintain the relationship between ecosystem services and human well-being [5]. Ecological security, which refers to the integrity and health status of ecosystem functions and services, especially natural and semi-natural ecosystems, is a prerequisite for sustainability and vital for the coordination of nature conservation and social development [6,7,8]. In the consideration of ecological security, some studies have focused on evaluating whether the regional ecosystem was within a safe range through indicators [9,10,11]. However, there are few specific planning schemes based on those indicators to improve ecological security. To satisfy the needs of planning and make up for this shortcoming, ecological security patterns (ESPs), which were proposed by Yu (1996), refer to the rational combination and layout of different landscapes according to the ecological process and the relationship between services, and form a spatial allocation plan to ensure the integrity of the structure and function of the ecosystem, which are significant for ecological security [12]. This has practical significance, as ESPs may provide methodological guidance for safeguarding ecological processes towards ecological security, which may contribute to regional sustainable development [13,14].

In general, ESPs consist of two parts: ecological sources and ecological corridors [15,16,17]. Ecological sources refer to momentous patches for ecological processes and act as the first step in constructing ESPs. Some nature reserves and important ecological regions have been directly regarded as ecological sources [18,19], and some indicator systems and ecological models (e.g., ecological risks and sensitivity, ecosystem services, and landscape connectivity) are useful for selecting vital ecological regions [20,21,22]. In particular, the evaluation of ecosystem services is the widely adopted method for identifying ecological sources [23,24,25]. However, in recent studies, most of the identification procedures of ecological sources have been static. Considering the sustainability of regional development, temporal factors can be added for the identification of sources.

The resistance surface is the foundation for establishing ecological corridors, which is used to reflect the difficulty of species migration or the spatial transfer of ecological processes [26,27]. The value of the resistance surface was commonly assigned by land types [28,29]. Due to this method of ignoring the internal differences of similar land types, some studies used habitat quality to construct a resistance surface by considering the biodiversity and compensating for the mentioned defect [16,30].

Ecological corridors are the carriers of species or ecosystem service flows in ESPs, and the minimum cumulative resistance (MCR) model has been most widely used to extract ecological corridors [31,32,33]. However, some shortcomings exist that only characterize the direction of ecological corridors and neglect other features (e.g., random migration of species, corridor width, and pinch points). Meanwhile, corridors can have clear boundaries, such as roads and rivers, or they can be invisible and composed of ecosystem service flows. Thus, circuit theory has been introduced to simulate the path of species migration in constructing ESPs [25,34]. Circuit theory with Ohm’s law has already been applied in landscape ecology science to explore the path of multispecies random walk and the landscape connectivity of the regions [35,36]. Accordingly, in the construction of ESPs, circuit theory can be applied to identify important corridors and the pinch points of these corridors [37,38].

In recent years, ESPs have been applied in species protection and city planning. On the one hand, to guarantee species-specific communication for information and connectivity, some scholars constructed ecological corridors using ESPs and graph theory [39,40,41]. On the other hand, rapid urbanization has resulted in serious landscape fragmentation, fragile ecosystems, and prominent contradictions between resources and the environment, and some scholars constructed city ESPs to ensure the healthy development of cities [42,43,44]. However, for relatively isolated nature reserves, where ecosystem services could benefit the surrounding areas, the discussion of ESPs is still insufficient; in particular, whether enlarging the protecting pattern of a nature reserve can obviously enhance ecological security remains an obscure goal for planners.

Wenchuan is the administrative unit of the Wolong Nature Reserve, which is an important panda reserve in China. Large-scale construction after earthquakes enhanced human livelihoods, but there could be a conflict in the consideration of natural conservation [45]. To promote interspecies communication and the external supply of ecosystem services, this study explored the scenario of ESPs in nature reserves and the surrounded areas, which aims towards guaranteeing regional landscape sustainability. We constructed ESPs through the following steps: (1) understanding the ecological security condition in terms of landscape transformation; (2) extracting ecological sources based on ecosystem services, especially the dynamic of ecosystem services, and mapping the resistance surface based on habitat quality; (3) constructing the ecological corridors based on circuit theory in nature reserves and in the surrounding area; and (4) comparing the parameters in ESPs based on the scenario of whether to include the surrounding area in nature reserves.

## 2. Materials

### 2.1. Study Area

**Figure 1:**
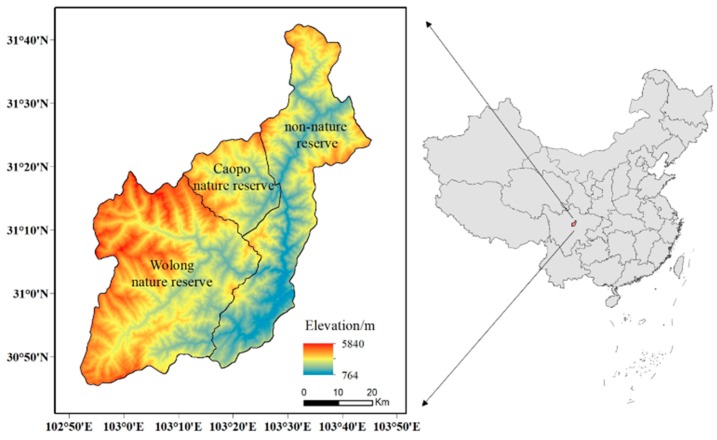
The location of Wenchuan.

Wenchuan is located in central Sichuan Province, which is characterized by a typical alpine canyon topography, and the vertical zonality of the climate is obvious. Thus, there is high diversity of species here, including pandas, which are one of the most precious species in the world. The area includes two nature reserves primarily set aside to protect pandas: the Wolong and Caopo Nature Reserves. The terrain gradually decreases from west to east, and most of the western mountains are above 3000 m. The reserves cover an area of 4084 km^2^ and include eight towns and four townships, and the majority of towns are distributed mainly in the eastern part of Wenchuan (Figure 1).

China is implementing policies to protect biodiversity and ecosystem services, and nature reserves are regarded as the chief approach to safeguard biodiversity and ecosystem services. In China, Wenchuan is an important area for panda protection and is a region with rapid economic development in Sichuan Province, and the conflict between human livelihood and natural protection is obvious. Therefore, ESPs need to be constructed according to the concept of sustainable landscape development.

### 2.2. Data Acquisition and Pretreatment

The data and their sources involved in the research are listed in Table 1. Due to the lack of evapotranspiration (ET) data in 2000 and net primary production (NPP) data in 2015, the starting year of ET was taken as 2001, and the terminating year of NPP was taken as 2014. The average monthly precipitation was based on the national meteorological site interpolated by thin-plate spline from 2000 to 2015. Because Wenchuan is only a county-level region, the spatial resolution of all data was resampled by cubic interpolation to a 30 m resolution, except for the data of land type and digital elevation model (DEM). The towns refer to the most basic administrative organ of 12 townships in Wenchuan and the road was regarded as the highway.

## 3. Methods

**Figure 2:**
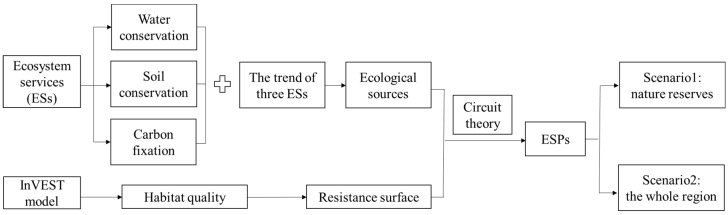
Flowchart on identifying ecological security patterns (ESPs).

The construction of ESPs mainly involved three stages. First, ecological sources were obtained through the evaluation of ecosystem services and their changes over the past 15 years. Resistance surfaces were mapped by assessing habitat quality. Then, combined with the ecological sources and the resistance surface of nature reserves, circuit theory was used to determine the corridors and the ecological pinch points that play an important role in the corridors. Finally, the ESPs of Wenchuan County were identified by introducing the additional ecological sources in non-nature reserves and adopting the whole area as a resistance surface. The flowchart is shown in Figure 2.

### 3.1. Identification of Ecological Sources

Ecological sources are the main ecological land with good habitat conditions, which can ensure regional ecological security and provide necessary ecosystem services. Owing to regional natural conditions and biological needs, we selected three essential ecosystem services: water conservation, soil conservation, and carbon fixation. Among them, water is the basic resource for organism survival, so water conservation assessment has become an important part of studies on ESPs [25]. Wenchuan County is located in a typical canyon topography, and we cannot neglect to assess the importance of soil conservation. Also, as the net amount of solar energy converted to plant organic matter, carbon fixation represents the primary food energy source [46]. The three ecosystem services correspond to water security, soil security, and so on. Based on these indispensable ecosystem services, the extracted ecological sources can be understood as the minimum demand for ecological land but can provide a larger ecosystem service supply.

According to the ecosystem services in 2015 and the trend of the ecosystem services from 2000 to 2015, the ecological sources were obtained. As shown in Table 2, three ecosystem services were assessed, namely, water conservation, soil conservation, and carbon fixation. Water conservation evaluation was realized by the principle of water balance, which is calculated according to factors such as soil thickness, permeability, terrain, and the flow coefficient. Soil conservation evaluation is mainly calculated by the revised universal soil loss equation (RUSLE) [47,48]. Considering that large-scale human engineering measures cannot be characterized, the factor of soil conservation measures (P) was assigned as 1. NPP was applied as a substitute for carbon fixation.

Moreover, a Mann–Kendall (MK) test with the Theil–Sen (TS) procedure was introduced to analyze the change trend of ecosystem services [56,57]:

(1) $$slope=Median\left(\frac{X_{j}-X_{i}}{t_{j}-t_{i}}\right)$$

(2) $$S=\overset{n-1}{\underset{i=1}{\sum }}\overset{n}{\underset{j=i+1}{\sum }}sign\left(x_{i}-x_{j}\right)$$

(3) $$sign\left(x_{i}-x_{j}\right)=\{\begin{array}{ccc} 1 & , & x_{i}-x_{j}<0\\ 0 & , & x_{i}-x_{j}=0\\ -1 & , & x_{i}-x_{j}>0 \end{array}$$

(4) $$Z=\{\begin{array}{ccc} \frac{S-1}{\sqrt{Var\left(S\right)}} & , & S>0\\ 0 & , & S=0\\ \frac{S+1}{\sqrt{Var\left(S\right)}} & , & S<0 \end{array}\;.$$

A slope > 0 indicates an increasing trend; a slope < 0 represents a decreasing trend; n is the length of the time series, and x_i_ and x_j_ are ecosystem services at times i and j, respectively; σ is the standard deviation; and |Z|> 1.64 was regarded as a significant change.

The change trend of three ecosystem services from 2000 to 2015 were divided into five grades: significantly decreased (assignment is −2), decreased (assignment is −1), unchanged (assignment is 0), increased (assignment is 1), and significantly increased (assignment is 2). We normalized the layers of these three trends and summed them to the normalized layers of each ecosystem service in 2015. In order to safeguard the ecological sources’ stability and to better agglomeration, the ecosystem services should be high and the landscape fragmentation should be low. Thus, after dividing the combined layer into five quintiles, the first 20% were extracted as ecological sources based on the Pareto principle (the 20–80 rule).

### 3.2. Ecological Corridor Extraction

#### 3.2.1. Resistance Surface

To deliver ecosystem services (i.e., water conservation, soil conservation, and carbon fixation) through corridors, the resistances of ecological flows should be considered. The resistance surface refers to the difficulty of species to pass through different landscapes [27], which would interfere with the ecosystem service flows. We took the nature reserve as the initial study area, which protects cherished wild animals and plants, and chose habitat quality to represent biodiversity and to construct the ecological resistance surface. Considering the special topographic factors in the study area, habitat quality is a combination of biodiversity threats and sensibility as determined by InVEST [58,59].

(5) $$Q_{xj}=H_{j}\left(1-\left(\frac{D_{xj}^{z}}{D_{xj}^{z}+K^{z}}\right)\right)$$

(6) $$D_{xj}=\overset{R}{\underset{r=1}{\sum }}\overset{Y_{r}}{\underset{y=1}{\sum }}\left(\frac{W_{r}}{\sum_{r=1}^{R}W_{r}}\right)r_{y}i_{rxy}\beta_{x}S_{jr}$$

where $Q_{xj}$ is the habitat quality of grid x in land type j, *H_j_* is the habitat quality score that ranges from 0 to 1, $D_{xj}$ is the total threat level of grid x in land type j, R is the number of ecological threat factors, *Yr* is the set of grid cells on r raster map, *Wr* is the threat weight, *r_y_* is raster map r, *i_rxy_* is the distance function of habitat quality and ecological threat factors, *β_x_* is the level of accessibility in grid cell x, *S_jr_* is the sensitivity of land use type j, and K and Z are scale factors.

The study area mainly protects species such as pandas; thus, the habitat quality tended to consider the pandas’ habitat quality. Considering the terrain and coexistence of nature and non-nature reserves, threat factors included slope, important towns, and general towns. Among them, in order to distinguish the impact of urban population, we regarded more than 4000 people as important towns, and the others as general towns.

The threat factor and sensitivity attribute tables are as follows (Table 3 and Table 4).

#### 3.2.2. Corridor extraction

Corridors are important landscapes for connecting ecological sources to guarantee species movements, genetic flows, or ecological flows, especially ecosystem service flows. Circuit theory based on Ohm’s law and graph theory were used to evaluate landscape connectivity, which contains various elements with ecological significance (Braaker et al., 2014). For example, ecological sources are regarded as the node of the circuit, the area of complex landscape is the resistance of different resistance values in the circuit, and the possibility of species migration in the region is the current, measured in amperes (A) [34].

According to the Linkage Mapper toolkit in ArcGIS and the resistance surface and identified sources, the minimum cumulative cost distance that referred to the path with the least resistance from multiple paired sources was calculated to determine the corridor direction. Then, based on the setting of the corridor resistance threshold, Pinchpoint Mapper was used to determine the current that reflected the net number of times of species or energy flows reaching the target habitat through the corridor in the ecological process passing through the corridor. If the current in the corridor is high, it can be considered to be an important corridor. A higher current in a specific area of the corridor indicates a pinch point. Pinch points indicate that species have a higher frequency throughout the region and play an irreplaceable role in species migration. A change in this trait will have a greater impact on the landscape connectivity.

Wenchuan has two nature reserves—Wolong and Caopo Nature Reserves—which are adjacent to each other in the west of Wenchuan. They are less interfered with by humans and have better landscape connectivity. The number of corridors, their range and mean of current, and the locations of pinch points were compared in two scenarios: scenario 1 represents ESPs only in nature reserves; scenario 2 represents ESPs between nature and non-nature reserves. These scenarios were used to answer the question: Is it necessary to enlarge the protection areas and construct ESPs between nature and non-nature reserves to protect the pandas?

## 4. Results

### 4.1. Landscape Transformation

**Figure 3:**
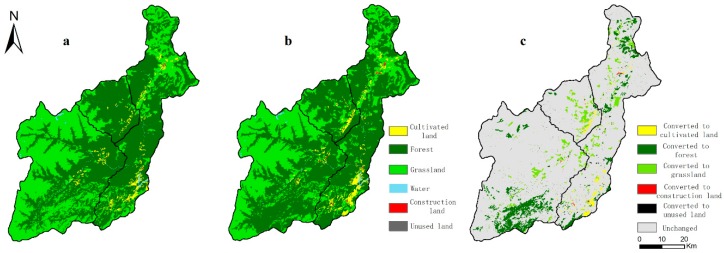
Land transformation from 2000 to 2015: (**a**) land type in 2000, (**b**) land type 2015, and (**c**) land transformation from 2000 to 2015.

Because the water bodies and unused land were basically unchanged, this study only analyzed the forest, grassland, cultivated land, and construction land during the period 2000–2015. In general, forest, cultivated land, and construction land have increased during the past 15 years, while grassland has decreased. Furthermore, the transformation was mainly concentrated in the southern part of the Wolong Nature Reserve and the areas where the rivers flow and the people gather. Specifically, the forest, cultivated land, and construction land increased by 11,221.83, 2034.63 and 94.32 ha, respectively. Grassland decreased by 13,958.83 ha. The area of forest increased obviously in the southern part of the Wolong Nature Reserve, while the forested area turned into grassland in the areas where rivers flow and people gather. The area of cultivated land continued to expand outward on the basis of the original location (Figure 3a–c).

**Figure 4:**
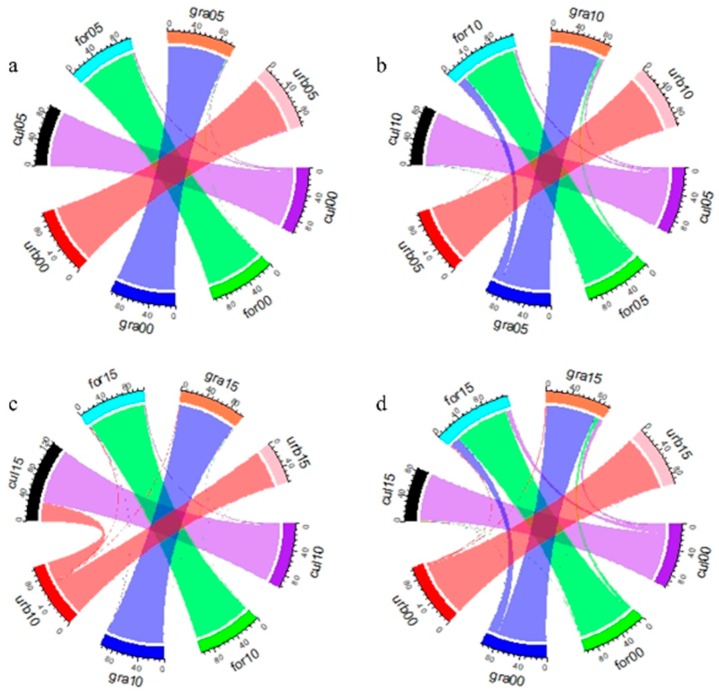
Change rate of different land types during 2000–2015: (**a**) 2000–2005, (**b**) 2005–2010, (**c**) 2010–2015, and (**d**) 2000–2015; urb, gra, for, and cul refer to urban, grassland, forest, and cultivated land; 00, 05, 10, and 15 represent 2000, 2005, 2010, and 2015.

The situation of every landscape transformation is displayed by change rate (Figure 4a–d). The results showed that the landscape transformation during 2000–2015 mainly occurred from 2005 to 2010. In general, the change rate of cultivated land, forest, grassland, and construction land was 17.5%, 8.1%, 17.4%, and 5.7% from 2000 to 2015, respectively. Among these data, the change rate of cultivated land, forest, grassland, and construction land was 6.4%, 1.4%, 0.1%, and 0% during 2000–2005, respectively, while it increased to 11.4%, 6.8%, 16.5%, and 1.92% from 2005 to 2010, respectively, and the cultivated land, forest, and grassland decreased to 5.4%, 1.8%, and 1.7% from 2010 to 2015. However, the change rate of construction land was 34.4% during 2010–2015, which was significantly higher than that of the two other periods. The “5/12” enormous earthquake in Wenchuan in 2008 [60] gave rise to a landscape pattern that changed greatly. After disaster reconstruction, the landscape was adjusted, especially for construction land.

### 4.2. Extraction of Ecological Sources from Ecosystem Services

**Figure 5:**
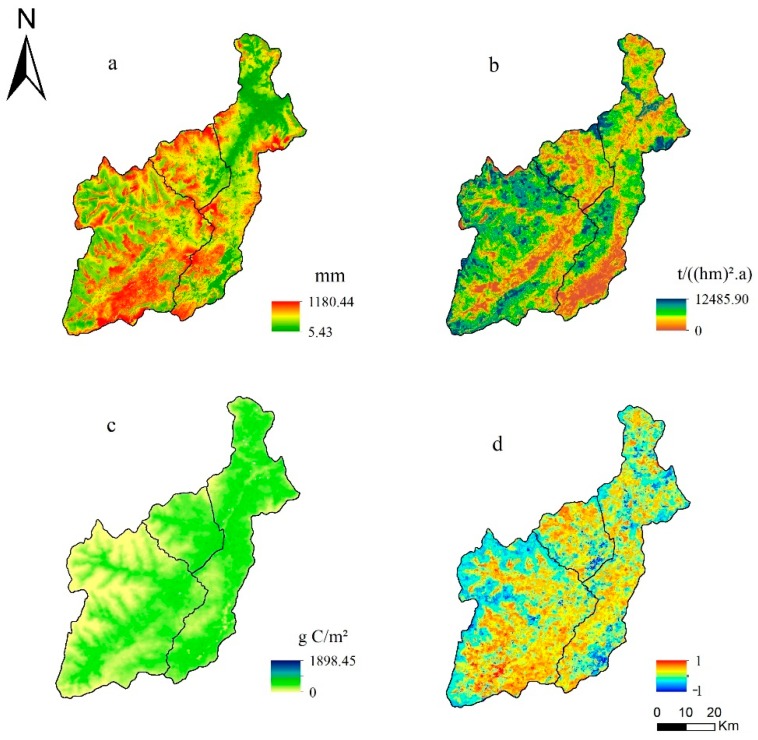
Spatial distribution of ecosystem services in 2015 and the trend during 2000–2015: (**a**) water conservation in 2015, (**b**) soil conservation in 2015, (**c**) carbon fixation in 2015, and (**d**) the trend of three ecosystem services during 2000–2015.

The high value of ecosystem services was mainly distributed in the Wolong Nature Reserve and the middle of non-nature reserves in 2015 in Wenchuan. Specifically, the average value of water conservation was 290.2 mm in the whole region. The average value in Wolong and Caopo (i.e., 312.2 and 310.9 mm) was higher than that in non-nature reserves (i.e., 254.6 mm) (Figure 5a). For soil conservation, the average value was 1156.83 t/(hm^2^·a) in Wenchuan, and the average value was 1293.3 t/(hm^2^·a) in Wolong. However, the average value was 1036.3 t/(hm^2^·a) in non-nature reserves, and it was 56.8 t/(·a) higher than the mean of Caopo (Figure 5b). At the same time, the average value of carbon fixation was 415.4 g C/m^2^ in Wenchuan. The average value of carbon fixation in non-nature reserves (i.e., 501.2 g C/m^2^) was higher than that in nature reserves (i.e., Wolong was 340.7 g C/m2; Caopo was 451.8 g C/m^2^) (Figure 5c). Thus, we need to consider establishing sources in non-nature reserves.

The results normalized the trend of integrated change of three ecosystem services, the scope of −1 to 1 (i.e., −1 was obvious degradation, and 1 was significant increase), which reflected the dynamic changes of ecosystem services in the past 15 years (Figure 5d). These ecosystem services can maintain a sustainable state, which is mainly located in nature reserves and the middle of non-nature reserves, except for the high-altitude and human-intensive regions. Specifically, these ecosystem services have significantly increased and are mainly distributed in forests, which are relatively concentrated, especially in the southern part of the Wolong Nature Reserve and in the western part of the Caopo Nature Reserve. However, they have experienced obvious degradation in the western Wolong Nature Reserve, the eastern Caopo Nature Reserve, and the northern and southern non-nature reserves.

Consequently, the ecological sources are mainly concentrated in nature reserves with low-altitude areas and in the middle of non-nature reserves. Furthermore, the forest is the main land type in the ecological sources in Wenchuan. To ensure the sustainability of ecosystem service supply, we used the 20% quintile to select ecological sources and the area of sources was more than 1000 ha.

### 4.3. ESPs

#### 4.3.1. The ESPs of Nature Reserves

**Figure 6:**
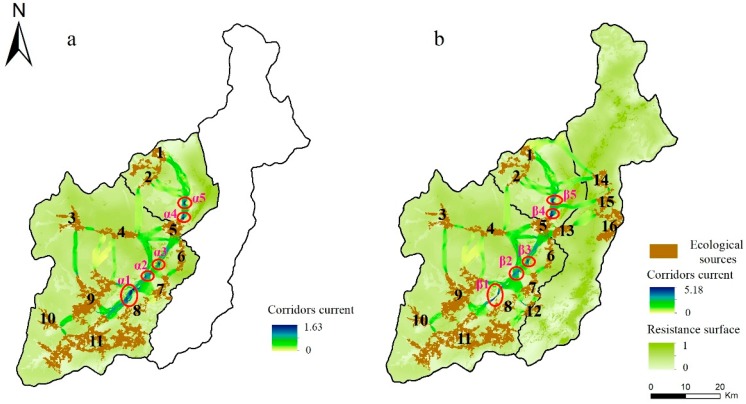
ESPs in two scenarios in 2015. (**a**) Scenario 1: ESPs in nature reserves; the location of pinch points: α1–α5. (**b**) Scenario 2: ESPs between nature and non-nature reserves; the location of pinch points: β1–β5.

In scenario 1, we selected 11 ecological sources accounting for 12.952% of the nature reserves, which are mainly concentrated in the eastern and southern parts of the Wolong Nature Reserve. The scenario indicates that approximately 12.2% of the area can meet the supply of the regional ecosystem services. The average resistance is 0.332, which indicates that the resistance of species (especially pandas) migration or ecosystem service flows is relatively small. The area contains 21 corridors, the average length of the corridors is 8386.364 m, and the results show that 21 pieces with a length of 8.386 km can maintain regional ecological security. The pieces were mainly located at lower elevations, especially to the east of the nature reserves where the forest was highly concentrated. At the same time, they constituted the high cumulative current value within the blue region (i.e., α1–α5). Due to the length of the corridors, the corridor between any two ecological sources forms a public corridor along sources 1, 2, 4–6, 7–9, and 11, which can be regarded as a corridor that play an important role in the landscape connectivity of the region, especially in the α1–α5 regions, which is vulnerable to becoming a “bottleneck” area that affects the landscape connectivity of the overall region (Figure 6a).

#### 4.3.2. The ESPs between Nature and Non-Nature Reserves

Based on scenario 1, ESPs between the nature and non-nature reserves were constructed in scenario 2. We added sources 12–16. Sources 14–16 are aggregately located in the middle of non-nature reserves, and they are important supply areas for ecosystem services for people in non-nature reserves. The area of ecological sources increased by 7489.081 ha, thus accounting for 9.884% of the total area of the region. Compared with the nature reserves, the average resistance of the non-nature reserve was larger, which increased the overall resistance value by 0.053.

Due to the increase of ecological sources in non-nature reserves, the number of ecological corridors increased by 10, with an average length of approximately 7617.968 m. Meanwhile, in comparison with scenario 1, the frequency of species (especially pandas) migration or ecosystem services flows increased by 0.01, 0.036, and 0.005 A in Wolong, grassland, and non-nature reserves, respectively. Thus, the results show that ESPs outside nature reserves can improve species (especially pandas) migration or ecosystem service flows and provide better ecosystem services to human-intensive areas on both sides through ecological sources in the middle of non-nature reserves. Furthermore, the region of pinch points includes β1–β5. The increase of average resistance in the whole region will affect species (especially pandas) migration or ecosystem service flows in ecological corridors, which will lead to a narrowing of the pinch points, especially in β1. Moreover, the locations of the pinch points have not changed, as they are still located in the low elevation areas on the right side of the junction of roads and water systems in the nature reserve (Figure 6b; Table 5).

Consequently, it is important to construct ESPs between the nature and non-nature reserves. Although this planning (scenario 2) increases the resistance of species (especially pandas) migration or ecosystem service flows in whole region, it increases the probability of random migration of species (especially pandas) in each region. The increase of ecological sources, especially in the central part of the unprotected areas, not only facilitates the connection between nature reserves and unprotected areas but also facilitates the supply of ecosystem services to the northern and southern parts of non-nature reserves where the population is concentrated. At the same time, the low-altitude area in the eastern part of the nature reserve is an important area for maintaining the connectivity of the whole region, and management and protection should be strengthened.

## 5. Discussion

### 5.1. ESPs for Landscape Sustainability

Based on the ideology of landscape sustainability and the approaches of ESPs, this article proposed methodological references for landscape sustainability, which can be implied as sustainable landscape patterns for both maintaining biodiversity and considering ecosystem services. This method includes two features: sustainable ecological sources and sustainable ecological corridors. Among them, the goal of ESPs is to ensure regional ecological security and improve the dynamic balance of the relationships between people and nature in terms of ecological sources and corridors. Meanwhile, the concept of landscape sustainability refers to the ability of the landscape to chronically maintain its basic structure and provide ecosystem services when the natural and social environment changes [61].

On the one hand, sustainable ecological sources consist of important ecosystem services with their dynamic changes. We regard the high-quality and relatively aggregated ecological function regions as ecological sources that can provide abundant ecosystem services and maintain the sustainable development of ecosystem services. Thus, we chose three ecosystem services that are important for protecting the ecological security of species (especially pandas) habitats and providing important water, soil, and oxygen services to humans, including water conservation, soil conservation, and carbon fixation, which are regarded as the indicator systems of ecological source extraction. At the same time, to ensure the sustainable development of ecological sources, we also added a dynamic analysis of these three ecosystem services to ecological source extraction.

On the other hand, sustainable ecological corridors not only enhance the landscape connectivity of the area by satisfying the ecological demand in the region but also improve the resistance and resilience of the ecosystem, as well as protect the biodiversity and sustain the ecosystem services. Based on circuit theory with the randomness of species migration and Ohm’s law, we proposed sustainability corridors. Compared with the traditional method of building a corridor MCR model, adding circuit theory on the basis of the MCR model can identify important corridors and priority protection areas (pinch point areas), thus making the ecological corridors more ecologically significant and more coincident with the concept of sustainable development, so that regional protection schemes can be formulated efficiently and the sustainable development of the region can be guaranteed in time.

### 5.2. Application of ESPs in National Park Planning

China’s national parks were established to protect the authenticity and integrity of the ecosystem and cultural heritage, and they also provide for the comprehensive functions of scientific research, education, and recreation [62]. However, plenty of national park pilots were based on nature reserves and scenic spots that integrated more protected areas and surrounding areas [63] but lacked certain methodological guidance. The nature reserves have not made effective plans for the simultaneous protection of both biodiversity and ecosystem services [64]. Therefore, how to guide the national parks to pursue green development under the national park system has become an urgent problem to solve.

In the context of building national parks, this paper suggests that the concept of sustainable landscapes can be used to identify sustainable ecological sources and construct sustainable ecological corridors to formulate sustainable landscape patterns for national parks, specifically by using indicators of ecological importance (e.g., ecosystem services, ecologically fragile areas, and landscape connectivity) to identify additional areas for national parks and regard them as ecological sources. Then, the ecological corridors are constructed by combining circuit theory with MCR or other methods to determine the least-cost corridors, which safeguards the national parks’ landscape connectivity and ecological security as well as the feasibility of implementation. This approach can also provide methodological support for the rational planning of landscapes in national parks.

Considering the complexity of ecological processes and the heterogeneity of spatial structures, there are some uncertainties in the quantitative results of this study, such as the construction of the resistance surface and the setting of the corridor width. On one hand, some parameters of habitat quality have been assigned subjectively in the resistance surface. In addition, due to the regional boundary being far away and thus threatening sources, the resistance value was relatively small, which led to the appearance of corridors at the boundary. Improved methods are required to specify resistance values and analyze their influence to formulate a comprehensive resistance value. On the other hand, owing to the threshold of cumulative cost distance increasing, the width of the corridors will be wider. However, wider corridors are not necessarily an advantage because they need to integrate conservation objectives, species behavior, human livelihoods, and actual situations. If these obstacles are addressed, ESPs can better methodologically support the practice of national park planning.

## 6. Conclusions

Based on the spatial relationship between ecosystem services and habitat quality in nature and non-nature reserves, a sustainable landscape pattern was constructed. The change of landscape change and ecosystem services mainly appears in the southern part of the Wolong Nature Reserve and roads. The ecological sources are mainly aggregated in the nature reserves, covering an area of more than 1000 ha; the high value of cumulative resistance distance was mainly in the area with roads or high elevation. By analyzing the two scenarios and comparing the area and ecosystem services in Wolong, Caopo, and non-nature reserves, increasing the area of the nature reserve in non-nature reserves can effectively improve the connectivity of the region and further protect the habitat quality of pandas. Meanwhile, the low-altitude area in the eastern part of the nature reserve is an important ecological protection site in Wenchuan. By reasonably planning ESPs in conflict areas of natural and economic development, the results of this study may contribute to providing a theoretical reference for the construction of national parks and regional sustainable landscape patterns.

## Figures and Tables

**Table 1:** Data types and sources.

Data	Data Sources	Resolution
Land type	The Resource and Environmental Science Data Center of the Chinese Academy of Sciences (http://www.resdc.cn)	30 m
Digital Elevation Model (DEM)	The Geospatial Data Cloud of the ASTER GDEM products (http://www.gscloud.cn/search)	30 m
Enhanced Vegetation Index (EVI)	The Geospatial Data Cloud of the MODEV1M products (http://www.gscloud.cn/search)	250 m
Evapotranspiration (ET)	The US Geological Survey (USGS) website of MODIS image’s MOD16A3 products	500 m
Carbon Fixation	The US Geological Survey (USGS) website of MODIS image’s MOD17A3H products	500 m
Soil Data	FAO’s HWSD 1.2 Global Soil Assimilation Database	1000 m
Monthly Precipitation Data	The National Meteorological Information Center (http://data.cma.cn/)	Vector
Road and Town Location	Baidu Point of Interest (POI)	Vector

**Table 2:** Ecosystem services calculation methods.

Ecosystem Services	Calculation Methods	Description
Water conservation	*WC* = *min*(1249/*V*) × *min*(1,0.9 × *D*/3) × *min*(1, $K_{\mathrm{soil}}$/300) × *Y* [49]	WC is the average annual water conservation (mm); V is the flow coefficient (using the data of model parameter table); D is the terrain index (digital elevation model);$\;K_{soil}$ is the soil saturated hydraulic conductivity (cm/d); Y is the water production [50]
Soil conservation	*Ar = Am − A* *A = R∙K·L·S·C·P* [47,48]	Ar is the soil conservation (t/($hm^{2}$·a)); Am is the potential soil erosion; A is the actual soil erosion (t/($hm^{2}$·a)); R is a factor of rainfall erosion (MJ·mm/($hm^{2}$·h·a)) [51]; K is a factor of soil erosion (t·h/(MJ·mm)) [52]; L is a factor of slope length [53,54]; S is the slope degree factor [55]; C is a factor of crop cover and management; P is a factor of soil conservation measures
Carbon fixation	Characterized by annual net primary production (NPP) in MOD17A3H products	The unit of measurement is g C/m^2^

**Table 3:** Threat factor attributes.

Threat Factors	Weight	Maximum Distance of Influence (km)	Decay
Road	0.6	3	Linear
Important town	1.0	6	Linear
General town	0.8	6	Linear
Cultivated land	0.3	2	Linear
Slope > 35°	0.7	3	Linear

**Table 4:** Sensitivity of different land types to different threat factors.

Land Type	Habitat Score	Road	Important Town	General Town	Cultivated Land	Slope > 35°
Cultivated land	0.30	0.50	0.60	0.90	0.00	0.80
Forest	1.00	0.80	1.00	1.00	0.70	0.40
Grassland	0.70	0.60	0.90	0.90	0.65	0.30
Water	1.00	1.00	0.80	0.85	1.00	0.70
Urban	0.00	0.00	0.00	0.00	0.00	1.00
Unutilized land	0.00	0.00	0.00	0.00	0.00	0.00

**Table 5:** ESP information statistics in two scenarios.

Index Systems	Scenario 1	Scenario 2	Landscape Implication
Area of ecological sources (hm^2^)/the percentage of regional area (%)	32,813/12.952	40,302/9.841	Important supply areas for ecosystem services
Average of resistance	0.332 (0.334/0.331) *	0.385 (0.334/0.331/0.426) *	Resistance level of species (especially pandas) migration or ecosystem service flows
Average current density (A)	0.017 (0.017/0.018) *	0.022 (0.027/0.054/0.005) *	Probability of species (especially pandas) migration or ecosystem service flows
Number of ecological corridors	21	31	The basic framework of maintaining regional ecological security
Average corridor length (m)	8386.364	7617.968	The difficulty of constructing corridors
Pinch point areas	α1–α5	β1–β5	High-frequency zone of species (especially pandas) migration or ecosystem service flows

* Remarks: (Wolong Nature Reserve/Caopo Nature Reserve/non-nature reserves).
